# Time-Use Sequences: A Mixed-Methods Study Exploring How, When, and Where Spatiotemporal Patterns of Everyday Routines Can Strengthen Public Health Interventions

**DOI:** 10.3390/ijerph21091128

**Published:** 2024-08-27

**Authors:** Brittany V. Barber, George Kephart, Michael Vallis, Stephen A. Matthews, Ruth Martin-Misener, Daniel G. Rainham

**Affiliations:** 1 Faculty of Health, Dalhousie University, 5968 College Street, Halifax, NS B3H 4R2, Canada; 2 Department of Community Health and Epidemiology, Dalhousie University, 5790 University Avenue, Halifax, NS B3H 1V7, Canada; george.kephart@dal.ca; 3 Department of Family Medicine, Dalhousie University, 1465 Brenton Street, Suite 402, Halifax, NS B3J 3T4, Canada; tvallis@dal.ca; 4 Department of Sociology & Criminology, The Pennsylvania State University, 211 Oswald Tower, University Park, PA 16802, USA; sxm27@psu.edu; 5 School of Nursing, Dalhousie University, 5869 University Avenue, Halifax, NS B3H 4R2, Canada; ruth.martin-misener@dal.ca; 6 School of Health and Human Performance, Dalhousie University, 6230 South Street, Halifax, NS B3H 4R2, Canada; daniel.rainham@dal.ca; 7 Healthy Populations Institute, Dalhousie University, Halifax, NS B3H 4R2, Canada

**Keywords:** spatiotemporal contexts, behavior change intervention, physical activity, geo-ethnography, mixed methods, time-use patterns, cardiovascular risk factors

## Abstract

Background: Behavior change interventions are critical for the secondary prevention of cardiovascular disease and for reducing the risk of a repeat event or mortality. However, the effectiveness of behavior change interventions is challenged by a lack of spatiotemporal contexts, limiting our understanding of factors that influence the timing and location in which day-to-day activities occur and the maintenance of behavior change. This study explored how behavior change interventions could incorporate spatiotemporal contexts of patient activities for modifying behaviors. Methods: A mixed-methods approach with adapted geo-ethnography techniques was used to solicit detailed descriptions of patients’ day-to-day routines, including where, when, and how patients spend time. Data were gathered from patients in one cardiac intervention program in Nova Scotia, Canada, from June to September 2021. Results: A total of 29 individuals (19 men and 10 women) between the ages of 45 and 81 and referred to the program after a cardiac event participated. The results show three key findings: (1) most patients exceeded the minimum guidelines of 30 min of daily physical activity but were sedentary for long periods of time, (2) patient time-use patterns are heterogenous and unique to contexts of individual space-time activity paths, and (3) time-use patterns reveal when, where, and how patients spend significant portions of time and opportunities for adapting patients’ day-to-day health activities. Conclusions: This study demonstrates the potential for interventions to integrate tools for collecting and communicating spatial and temporal contexts of patient routines, such as the types of activities that characterize how patients spend significant portions of time and identification of when, where, and how to encourage health-promoting changes in routine activities. Time-use patterns provide insight for tailoring behavior change interventions so that clinic-based settings are generalizable to the contexts of where, when, and how patient routines could be adapted to mitigate cardiovascular risk factors.

## 1. Background

Secondary and tertiary behavior change interventions for cardiovascular disease are critical for the management of risk factors and the prevention of future cardiac events [1]. Cardiovascular disease (CVD) (including both ischemic and cerebrovascular disease) is a leading cause of death in the United States and Canada and is responsible for more than 30% of all deaths worldwide [2,3,4]. CVD is largely preventable, and interventions focused on sustained modification of high-risk factors through lifestyle and pharmaceutical therapy would significantly curtail the risk of CVD and improve population health [5,6,7]. Reducing the prevalence of risk factors associated with CVD, including high blood pressure, high cholesterol, poor nutrition, smoking, obesity, physical inactivity, and medication adherence, are critical even post-cardiac event to reduce the probability of a repeat event and mortality [4,6].

### 1.1. Advancements in Behavior Change Interventions

Characteristics of successful behavior change interventions that reduce the risk of CVD or a repeat event most often adopt a theoretical framework, incorporate strategies tailored to the individual needs of patients, and use technologies to support adherence to behavior change. Behavior change interventions that use a theoretically informed intervention design show improved outcomes for physical activity (PA) [8], weight loss, smoking cessation, and the management of hypertension, lipids, and blood glucose [9]. The most frequently cited theories within CVD interventions include Social Cognitive Theory, the Transtheoretical Model, and the Theory of Planned Behavior/Reasoned Action [8,9]. Effective behavior change interventions are also flexible enough to incorporate strategies tailored to individuals’ needs and characteristics [1,10,11]. For example, interventions that use a tailored multifaceted approach with educational resources, mobile text reminders, and individualized counseling from a case manager have been found to improve the reduction in CVD risk after a cardiac event [12]. Technology-based interventions have increased adherence to PA using mobile texts, gamified applications, wearable sensors, and telehealth counseling [9,13]. For example, the use of innovative gamified mobile applications within CVD interventions have been shown to improve self-management and knowledge of medications and reduce risk factors, like physical inactivity [14]. Most effective gamification features include elements of surprise/novelty, having teammates, individualized challenges, and stimulating graphic design [14]. While some interventions with these characteristics show improved effectiveness for a reduction in CVD risk, evaluating the effectiveness of intervention characteristics is complex due to varied design and outcome measures. There is continual debate as to whether interventions focused on single behaviors versus those focused on multiple behaviors are more effective in promoting health and reducing CVD risk [15].

### 1.2. Limitations of Behavior Change Interventions

Even with advances to improve intervention design (theoretical, tailoring, and technologies), interventions are challenged by poor maintenance of behavior change and limited information characterizing spatiotemporal contexts influencing the timing and location in which individual activities occur. For example, clinic-based interventions, in comparison to community-based interventions, are effective when being delivered within organized settings that provide regular contact with healthcare professionals and supportive peer-to-peer interactions. However, when an intervention ends, and patients return to the contexts of their day-to-day lives, they are challenged to maintain health-improving routines [1]. Patients are most challenged by day-to-day disruptions, preventing the development of stable routines [16], time management [17], and recall of intervention information [10,18] and diminishing motivation without regular contact with healthcare professionals and peers [19]. These challenges have been countered by tailoring individualized approaches [11,20]. However, it is less evident how most of these interventions are tailored to the contexts outside clinic-based settings that influence where, when, and how health activities occur. For example, PA prescriptions within clinic-based settings [21,22] use generic recommendations and are focused on individuals without accounting for the unique contexts of day-to-day activities that occur within environments (physical or social) that are sometimes beyond their control. Yet, we know that social and physical environments play an important role in promoting or inhibiting health behaviors [23], including contexts that influence the timing and location of day-to-day activities. Assessing the unique spatiotemporal contexts of patients’ day-to-day activities is critical for tailoring intervention strategies so that clinic-based settings are generalizable to the contexts around patients. 

### 1.3. Methods to Assess Contexts Influencing Behaviors

Different ways to assess contexts of health activities include time-use techniques, map-based questionnaires, accelerometers (and other activity monitoring sensors), global positioning system (GPS) data loggers, or combinations of techniques. Time-use measurements provide a way to examine temporal dimensions of activities, commonly through diaries or survey-based methods that identify patterns in activities of daily living [24,25]. For example, time-use diaries have been used to better understand how individuals adapted daily activities and routines in response to COVID-19 public health restrictions in the United Kingdom [26]. Time-use surveys have also been used to improve national surveillance of PA, like the American Time-Use Survey, assessing the amount of time people spend engaging in various activities, including paid employment, unpaid caregiving, and leisure [27]. Map-based questionnaires gather context through a combination of online mapping software for collecting locational coordinates of activities in addition to survey questions for gathering context [28,29]. For example, map-based questionnaires have advanced disease surveillance in rural areas of the Philippines and Indonesia by locating households of clinic attendees to estimate the magnitude and heterogeneity of malaria transmission where formal addresses are unavailable [30]. 

Wearable motion-sensing technologies, like accelerometers, have commonly been used to gather more reliable estimates of PA and sedentary activities [31]. For example, accelerometers have been useful for measuring the level of exertion according to cut points associated with metabolic equivalent scores (METs) for large-scale epidemiologic studies [32]. Wearable GPS data loggers can accurately record movement patterns and can assist in identifying the contexts and timing of health-related activities [33,34]. For example, GPS data have been used to estimate exposures to both health-promoting (e.g., interactions with green spaces) and health-harming (e.g., areas with poor air quality) contexts [35,36]. Innovative methods using a combination of the approaches mentioned above contribute to an enriched understanding of the contexts in which health-related behaviors occur. For example, GPS and accelerometer data have been used to contextualize supportive environments of PA for youth by identifying where, when, and how much PA they engage in [37]. Another method known as ecological momentary assessment (EMA) uses an electronic device to notify participants at fixed or random times throughout the day to respond to survey questions about when, where, what, and with whom behaviors occur [38,39]. EMA generates real-time information on participants’ behaviors, perceptions, and emotions when they occur in natural environments with repeated occurrence and observations of events over time [40]. For example, EMA has been used to explore daily patterns of park use, leisure time PA, and perceptions of stress [41]. EMA daily surveys indicate that when participants visited a park during the day, they reported less stress in the evening in comparison to participants who did not visit parks (OR = 0.67; 95% CI = 0.58–0.77), and when daily park use occurred with leisure time PA, participants reported less stress (OR = 0.58; 95% CI = 0.59–0.69) in comparison to participants engaging in only one of the two activities [41].

The previously mentioned approaches are effective for gathering information about where and when health behaviors occur; however, they are limited without information contextualizing why and with whom health behaviors routinely occur. To better understand factors that connect people, time, and place, a mixed-methods approach is required to gather contextual descriptions of what, when, why, and with whom activities occur [42,43]. Geo-ethnography is one mixed-method approach that couples geospatial and ethnography methods to explore how locations and the built environment contextualize the lived experiences of individuals [42]. The significance of mixed-method techniques, like geo-ethnography, is that they contribute to a comprehensive understanding of quantitative patterns of behavior with qualitative contexts of perceptions, experiences, and social relationships that influence why individuals make decisions about their activities [42,44]. For example, geo-ethnographic methods have been employed to explore how food insecurity affects low-income urban and rural women by exploring spatial patterns and contexts of food shopping behaviors [44]. A visualization of spatiotemporal patterns of health behaviors is important for tailoring intervention strategies by identifying opportunities or constraints for behavior change and supporting patients across diverse contexts.

The coupling of ethnographic data with geospatial techniques provides a different approach for contextualizing the complexity of patients’ daily activities and has not yet, to our knowledge, been applied within a behavioral intervention setting. Geospatial mixed methods provide an opportunity to strengthen the effectiveness of behavioral interventions by tailoring strategies based on information about patients’ spatiotemporal patterns of health behaviors [45]. In this paper, we explore the application of a mixed-methods approach known as geo-ethnography to gather spatiotemporal information about the contexts influencing patients’ day-to-day routines. 

The aim of this study is to explore how behavior change interventions can solicit information from patients about the spatiotemporal contexts in which their health activities occur and how this information can be used to influence the design of interventions. To achieve this aim, this study answers two specific research objectives as follows:

(1) Investigate spatiotemporal contexts about patients’ day-to-day health activities;
(2) Explore how interventions can use spatiotemporal information about patients’ day-to-day health activities to overcome challenges to physical activity within clinical interventions.

## 2. Methods

### 2.1. Study Design

**Figure A1:**
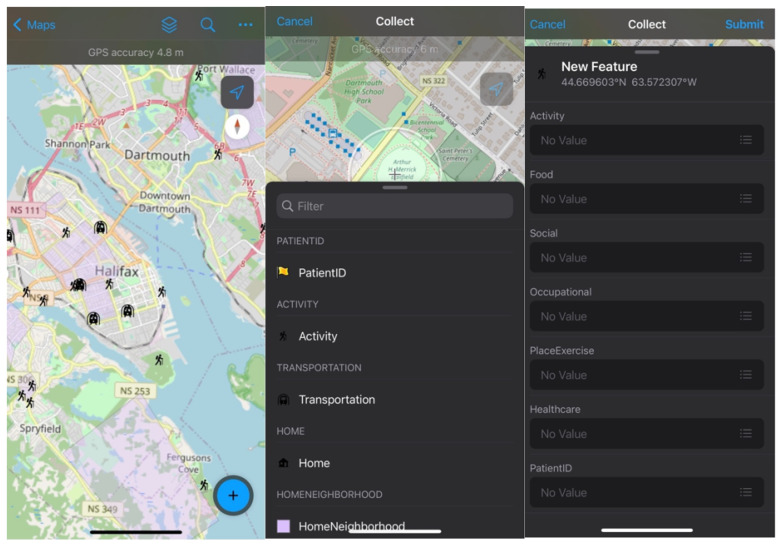
Field Maps Application Interface.

To solicit patients’ spatiotemporal patterns of health activities, we applied a mixed-methods approach to gather detailed descriptions of day-to-day routines, including where, when, and how patients spend their time. This study design was adapted from the geo-ethnographic methods proposed by Matthews et al. [42] by engaging patients in two semi-structured qualitative interviews supported by a digital mapping tool. The ESRI Field Maps application was used to create a customized digital mapping tool for capturing locations of patients’ activities during in-person interviews. The online mapping tool was developed for field data collection activities where data are geocoded and stored in a database for further analysis. This digital map tool was customized through an ArcGIS online account that was used to create individual layers of the features and fields for data collection (Table 1). Each layer was assigned a field value to further define the types of activities each patient described. Table 1 presents each layer’s name, feature class, field ID, and value ID that was assigned to patient activities during interviews. A visualization of the Field Maps application is included as supporting information (Appendix A Figure A1).

### 2.2. Geo-Ethnography Techniques

Geo-ethnographic techniques include visualizing routine activity patterns that interviews alone would not represent, such as locations of resources and different routes individuals must travel to navigate healthcare appointments, childcare, or commuting for employment [42]. Furthermore, an in-depth understanding of the contexts that influence activity patterns provides details about activities that maps alone would not represent, such as having fewer social supports, concern for neighborhood safety, or access to transportation [44]. To answer each research objective, first, this study explored a way of adapting geo-ethnographic methods to collect spatiotemporal contexts of patient health behaviors, and second, how this information could be used within an intervention as a data collection and communication tool to modify behavior change.

### 2.3. Research Setting

This study took place at a cardiac prevention and rehabilitation intervention program for patients at high risk for CVD or having recently experienced a cardiac event. Patients are referred to the program by a primary care provider or by hospital outpatient services after experiencing an event. The goal of cardiac prevention and rehabilitation intervention programs is to support patients with behavior change to improve nutrition and increase PA. Programs deliver a series of health education and PA classes that are led by a collaborative team of healthcare professionals, including a dietician, nurse practitioner, physiotherapist, and cardiologist. 

The cardiac program purposively selected as the research setting has a heterogeneous patient population that lives across urban and rural communities. Previously delivered in-person over 12 weeks, when data collection began in June 2021, the cardiac program was condensed to a 6-week hybrid model comprising in-person PA and virtual education classes due to COVID-19.

Prior to enrollment in the cardiac program, patients complete an intake assessment to gather baseline health measures from blood work, a PA stress test, medical records, and the current level of PA and eating habits. Patients attend a weekly 1 h virtual education class via Zoom and a 1 h in-person PA class. The education sessions focus on a range of health topics incorporating evidence-based guidelines for PA, nutrition, risk factors, stress, and medications. PA classes are structured as circuit training, with a 15 min warm up and cool down and a 30 min rotation between a stationary bicycle, treadmill, and weighted strengthening exercises. Patients receive an individualized bi-weekly phone call with a healthcare professional to discuss any questions or medical concerns. At the end of the 6-week program, patients complete a follow-up assessment to compare blood work, level of PA, and dietary habits. 

### 2.4. Patient Recruitment and Data Collection

Patient cohorts enrolled in the cardiac program were purposively selected to participate in this research study. Purposeful recruitment began with cohorts of 8 to 10 patients in week 2 or 3 of the 6-week program. Potential patients were first approached for recruitment by a healthcare professional who provided information about the purpose of the study and what would be required to participate. Patients who agreed to be contacted by the primary researcher provided their phone number and first name. The recruitment process was repeated with 5 different patient cohorts, recruiting 6 patients from each cohort, to gather a sample size sufficient for qualitative saturation [46]. To acknowledge the time commitment required for patients to be interviewed in this study, every patient was offered an honorarium in the form of a CAD 25 gift certificate to a grocery store.

Patients were interviewed in-person by the first author at the cardiac program between June and September 2021. Each patient participated in two interviews that were audio recorded. During the first interview, patients were asked to interact with the online mapping tool and identify the exact locations where they live and work and the types of activities that comprise their day-to-day routine. Participants were asked to describe their routine activities in a given week to depict typical routines. Interviews were structured to gather contextual information about patients’ typical time-use patterns, including where and when activities occurred, how much time was spent during each activity, and whether the activity was performed alone or with others. Patients were asked “How would you describe your typical routine on a given day, beginning with what time you wake up in the morning?” “What sorts of activities do you do in your neighborhood?” During the second interview, patients were asked to provide contextual information about their activities, including when and why routine activities occur, relationships that influence activities, and perceptions about adapting routine activities. Patients were also asked to discuss their health goals and factors that support or prevent them from reaching health goals. Interview questions included “What are some of your health goals you are working towards?”, “Are there times during the day you find it difficult to stay on track with your goals?”, and “How might your activities be influenced by who you are with?”

### 2.5. Ethical Approval

Ethics approval for this study was obtained from the Nova Scotia Health Authority (REB#1026722). All patients provided informed consent for their de-identified and anonymous personal health information and direct quotes to be included in the research results.

### 2.6. Analysis

Interviews were audio recorded, transcribed verbatim, and then coded by the first author with the aid of QSR NVivo12.6.1 qualitative data analysis software [47]. Interview transcripts were openly coded by grouping patient activities into categories of time use. The first author coded patient activities and time expenditure directly from interview transcripts so that the time and location of each activity could be stacked within a 24 h period to create a sequence of routine time use. Initial codes and time-use sequences were reviewed and discussed collaboratively by two members of the research team (MV, DR). Supplemental information on distances and locations was retrieved from data in Field Maps. Approximate commute times between two locations were verified using Google maps.

Geo-ethnographic mixed-method analyses have previously been used to develop map-based visualizations of spatiotemporal contexts where activities take place, including locations of points of interest, neighborhood boundaries, built environments, and routine distances traveled [42,44]. For example, MacNell (2018) [44] used geo-ethnographic methods to analyze quantitative data from patient interviews by geo-coding the locations of grocery stores and patients’ home addresses to calculate the distances patients traveled outside their neighborhoods to shop at a preferred grocery store. This study adapted geo-ethnographic analyses to develop a clinical tool that could visually represent important contextual information about what, when, where, and with whom activities occur.

Using quantitative data in Field Maps and qualitative interview data, the first author created map-based visualizations of patients’ routine activities. The research team held regular meetings to discuss ways of visually representing the sequencing of activity times and locations, including the contexts of what activities occurred at different locations, when and for how long activities took place, and with whom activities occurred. The research team deliberated the complexity and applicability of map outputs within clinic-based settings and discussed the challenges of interpreting map-based visualizations of patients’ day-to-day routines, such as limitations of identifying the sequences of time to depict when and for how long certain health activities occurred. The idea of representing time-use patterns within a stacked bar chart was reached by discussing how spatiotemporal data from geo-ethnographic techniques could be combined with time-use diaries [26] to represent the sequencing of timing and locations of patients’ day-to-day activities. A visualization of stacked bar charts was customized by ordering patient activities into a 24 h time sequence and then coding by location to depict unique geo-sequence patterns of behavior. 

## 3. Results

A total of 58 interviews with 29 patients (19 men and 10 women) were conducted. Two interviews were conducted with each patient, lasting on average 71 min (range of 37 to 110 min). A total of thirty-one individuals were recruited as potential patients; however, two dropped out of the study before the first interview due to lack of time to participate. A summary of patient characteristics is provided in Table 2. Almost all patients were between the ages of 45 and 81, married/common law, and had a college or university degree. Most patients were employed full time or retired. Approximately half of the patients were non-smokers or previous smokers, and only one patient was a current smoker. Patients were typically referred to the cardiac program after experiencing myocardial infarction. Two patients were referred to the program for prevention with no self-reported comorbidity. All patients completed the entire 6-week program.

Interviews were openly coded based on participants’ descriptions of activity type. Emergent activity categories included sleep, sedentary behavior, light PA, and MVPA (moderate to vigorous physical activity). Patients’ descriptions of activities were compared to existing evidence of activity category definitions and guidelines of PA [48,49,50]. Definitions of activity categories are summarized in Table 3. The first author completed the coding of data twice, 1 week apart, to ensure intra-coder reliability of the results [51]. The coding of time-use patterns was completed by manually entering time-use data into a Microsoft Excel spreadsheet to record the length of time and location of each activity that was performed for all patients. The results of the coding were reviewed by two researchers (DR, GK) and then compared to detect inaccuracies, and a final version of the validated results was used to create a stacked bar chart of patient routine activities. The stacked bar chart was manually edited within Excel to color code patient activity categories and provide labels with a corresponding legend to specify different types of activities.

### 3.1. Sequencing and Visualization of Time-Use Patterns

**Figure 1:**
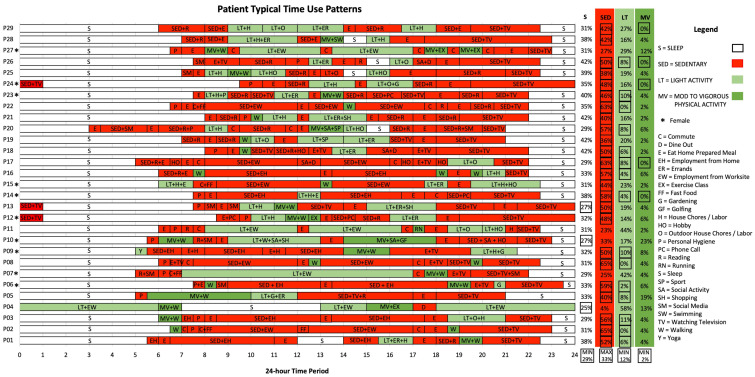
Typical time-use patterns.

The results from patients’ spatiotemporal patterns of health activities are presented visually as time-use patterns (Figure 1). A stacked bar chart was used to represent patients’ routine time-use patterns. The visualization of spatiotemporal sequences enables quick insight into the patterns in timing and location of health behaviors across patient groups, with details of individual differences in health behaviors between patients. A summary of the total time spent in each activity category was calculated for each patient, with boxes to indicate when patients’ activity levels were less than the minimum or greater than the maximum recommended guideline per day. Activity levels were converted to a percentage of 24 h. Commute times were accounted for when they could be estimated between two known locations; otherwise, irregular commute times were grouped within activity time. 

**Figure 2:**
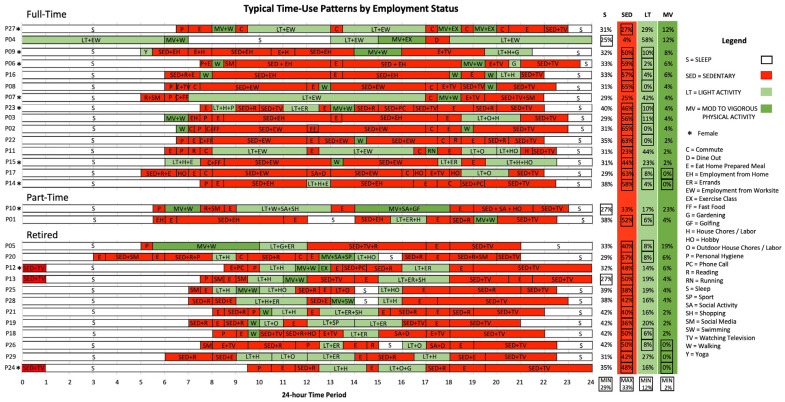
Typical time-use patterns by employment status.

**Figure 3:**
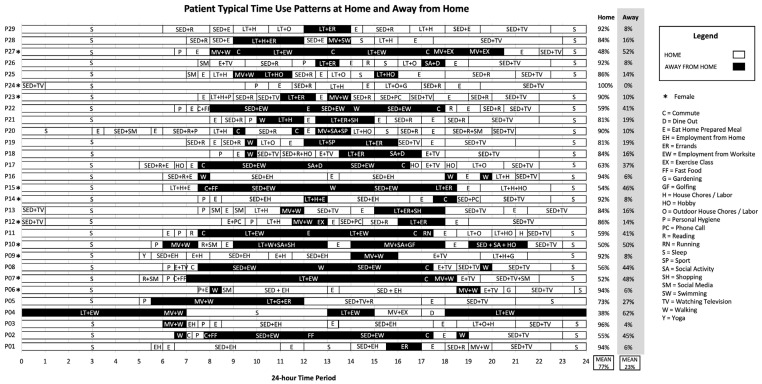
Typical time-use at home and away from home.

Three key findings are presented from spatiotemporal contexts of patients’ day-to-day health activities. (1) Almost all patients (*n* = 24) exceeded the minimum guidelines of 30 min of daily PA but were sedentary for long periods of time (Figure 1), (2) patients’ time-use patterns were heterogeneous and unique to contexts of individual space-time activity paths (Figure 2), and (3) time-use patterns revealed when, where, and how patients spend significant portions of time and opportunities for adapting patients’ day-to-day health activities (Figure 3).

### 3.2. What Are Spatiotemporal Contexts of Patients’ Day-to-Day Health Activities? 

Spatiotemporal contexts of patients’ day-to-day health activities are presented (Figure 1) and summarized (Table 4) according to emergent activity categories of sleep, sedentary, light, and MVPA. 

### 3.3. Spatiotemporal Characteristics of Patients’ Time-Use Patterns

Patients’ time-use patterns show that close to all patients (*n* = 24) engage in a minimum of 30 min of MVPA when walking alone in their neighborhood in the morning or the evening around dinner time. Patients self-identifying as female spent less time sedentary (mean F = 44 min, M = 47 min) and more time engaging in light PA (mean F = 16 min, M = 14 min) and MVPA (mean F = 6.5 min, M = 4.2 min) in comparison to self-identifying male patients. Few patients (*n* = 4) engaged in MVPA social activities with friends or family, such as exercise classes or sports, as part of their routine (Table 4). For example, when asked what sorts of activities patients do in their neighborhood, one patient describes “walking, I walk the dog every morning. All over the place… I’ll walk the dog first thing, between six and seven. That’s a typical Monday to Friday” (P02, M). Another patient describes their typical morning routine as follows: “I go for a three and a half hour walk every morning around my neighborhood. I start at 5:30 a.m. and finish around 9:00 a.m.” (P05, M). Patients commonly walk alone, as described by P10 as follows: “My walk that I do in the morning is almost every day for an hour and twenty minutes or so, it’s just under six kilometers. I usually go out alone around six or seven when it’s quiet” (P10, F). 

Despite achieving goals for a minimum of 30 min of MVPA, substantial amounts of time were spent in sedentary activities. Long stretches of red (Figure 1) represent sedentary activities occurring mid-day while patients were working from home or after dinner when patients watched television with family or were reading alone. Although P16 walks their dog for 30 min in the morning, afternoon, and evening, they are sedentary for more than half of their day (57%) while working, stating “well I work most of the day, so most of the day is sitting. I do computer stuff and so most of the day is on the computer” (P16, M). Further, most sedentary activities occurred during the evening when patients were at home, such as watching television alone or with family. P12 describes “I try to read at least an hour, and if there is nothing else to do, I could spend all afternoon reading. In the evening is when I watch TV, any time after seven until after midnight. It could be one o’clock until I go to bed” (P12, F). 

Periods of light PA occurred mid-day while at work, running errands, or shopping, with some light PA occurring at home for house chores, outdoor labor, or gardening. Patients engaging in light PA for work (P04, M; P07, F; P11, M; P27, F) spent, on average, six hours more than all other patients, reducing their sedentary activity time in half compared to patients with sedentary employment. P27 describes her employment routine as follows: “I work seven days a week. I do private homecare. So, I’m busy with that. Every day I have three clients, and I go there and make all their meals, do laundry, bathing, take them to all their appointments. And every night I go back between nine and ten to get them ready for bed and put them in bed” (P27, F). 

Almost all patients met the daily minimum guidelines for sleep every day. Of three patients who typically had less than the recommended seven hours of sleep, P04 had on average six hours of sleep due to remote shift work. P04 described “I work on the sea, so offshore life is twelve-hour watches. Being a senior officer, I’m never off duty. So, if you’re working twelve-hours and an emergency comes up, you have to get up. I’ve always worked a night shift” (P04, M). 

### 3.4. How Can Interventions Use Spatiotemporal Information about Patients’ Day-to-Day Health Activities?

Spatiotemporal characteristics of patients’ time-use patterns reveal that day-to-day health activities are heterogeneous and unique to the contexts of individual space-time activity paths. Although patients were participating in the same standardized intervention, they experienced very different space-time contexts (Figure 1). Findings from time-use patterns could be stratified to visualize differences in patient activities and used to overcome challenges to PA within clinical interventions. Stratifying time-use patterns by employment status (Figure 2) and where patients spend most of their time (Figure 3) highlight differences in space-time activity paths and opportunities for increased PA. 

### 3.5. Characterizing Heterogeneity of Patients’ Time-Use Patterns

By stratifying patients by MVPA and comparing patients employed full time, part time, and retired (Figure 2), we can better understand the characteristics of the types of activities they engage in, such as the timing and location of activities throughout their day. Stratifying typical time-use patterns by MVPA and employment status (Figure 2) shows variability between patients who were employed full time (*n* = 15), part time (*n* = 2), and retired (*n* = 12). Patients employed full time (M = 8; F = 7) had less variability in activities throughout their day and were sedentary for longer periods of time while working in comparison to patients who were employed part time (M = 1; F = 1) or retired (M = 10; F = 2) and were engaged in many light physical activities for shorter periods of time throughout their day. Patients employed full time engaged in less MVPA throughout their day, walking at the start or end of their day. P15 described “If I’m not intentional, I’m lucky if I get twelve hundred steps in a day, like I would have to be very intentional about moving” (P15, F). Whereas retired patients engaged in more MVPA and light activities throughout their day, including different activities like sports, swimming, running errands, and house chores/labor. P12 described “well I get up, I try to get my shower going, or wash the laundry or water the flowers. Somebody may call me but in between that, I like to get out for a walk. But if I’m going down to do my laundry, I’ll get on the bike, and wait until the cycle is over and I’ll hang the load of laundry” (P12, F). Although retired patients were more active throughout the day, they were sedentary for longer periods of time at the end of their day while watching television after dinner in comparison to patients who were employed full time and part time and engaged in chores. P28 described “I’ll have supper, and I’ll usually just kind of watch shows or sports on TV. I do have a bunch of shows that I record, and I watch them after they’ve been recorded. Yeah, I’m usually relaxing, watching TV. I don’t need things. I don’t do a lot of stuff” (P28, M). In comparison, P03 described their typical evening routine as follows: “I work until late afternoon, four or five, have dinner, and then do whatever needs to be done around the home. We’ve been doing some renovations, some yard work, that sort of thing” (P03, M).

### 3.6. Characterizing the Space-Time Continuum of Where Activities Occur

Stratifying time-use patterns by the space-time continuum of activities, such as activities occurring at home and away from home, could also help visualize differences between patient activities and overcome challenges to PA within clinical interventions. Stratifying typical time use at home and away from home (Figure 3) shows that patients spend most of their time at home (mean 77%, range 38–100%) and less time away from home (mean 23%, range 0–62%). Male and female patients self-reported similar time-use patterns at home (M = 77%, F = 76%) and away from home (M = 23%, F = 24%). 

Having patients visualize where and how they spend their time could increase awareness of opportunities to integrate PA throughout their day based on resources in different places and the benefits of increasing light PA to reduce sedentary time. P22 described opportunities for increasing PA at work, stating “based on my schedule, whether I have more conference calls or a break in my schedule, I put my earphones in and do a conference call while I walk” (P22, M). P17 described opportunities for increasing physical PA at home, stating “I plan on exercising at home. Since midway through this program, I’ve started to exercise at home because we have a treadmill at home and a bike. I never used to bother with it, but my wife does. But now I am, even for ten minutes every day in the mornings and sometimes at night as well” (P17, M). Visualizing where patients spend most of their time, such as at home or away from home, is important for understanding differences in space-time activity patterns of PA and sedentary activities across patient populations, including where and who patients are with and opportune time and place to overcome challenges to PA.

## 4. Discussion

The aim of this study was to explore how behavior change interventions could gather spatiotemporal information about the contexts influencing patients’ day-to-day routines and how this information could be used to overcome challenges to increasing PA by clinical interventions. Results show most patients exceeded the minimum guidelines of 30 min of daily PA by walking alone in their neighborhood but were largely sedentary while working or watching television for long periods of time day to day. Time-use patterns provide a visualization of patients’ unique geo-sequencing of activities [52,53], including important contextual information that influences how routines are developed day to day. Geo-ethnographic techniques were useful for better understanding heterogeneity between patients participating in the same intervention. Identifying heterogenous patterns in patients’ space-time activity paths is important for clinical interventions so that program design and education can be tailored to patients experiencing similar space-time activity patterns (i.e., working full time, part time, retired). Furthermore, identifying contexts surrounding patient day-to-day activities such as when, where, and how space-time activity patterns occur (i.e., time spent at home versus away from home) is important for identifying how clinical programs can work with patients to tailor context-specific strategies to the most opportune time and place for disrupting routines and modifying behavior change. Bringing awareness to the totality of activities and interactions with people and places over time, the use of time, and factors influencing decision making is critical for supporting the long-term maintenance of healthy routines. Geo-ethnography techniques provide a useful visualization tool for individuals who may not fully understand how to adjust their routine behaviors that have become automatic over time and healthcare professionals who may not be aware of how to adjust intervention techniques to encourage individuals to develop stable everyday routines. 

### 4.1. Clinical Relevance of Time-Use Patterns

**Figure 4:**
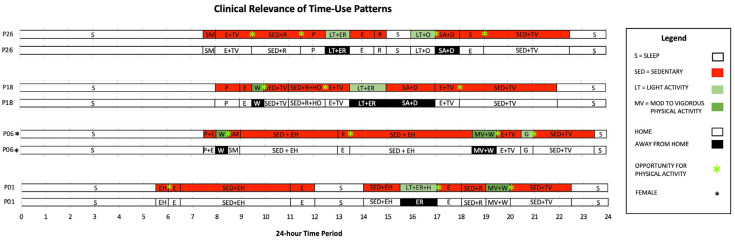
Opportunity for physical activity.

Intervention strategies, like prescribed PA, have been used in clinic-based settings for over 15 years [21] yet have shown limited effectiveness for sustained increases in PA [22,54,55,56]. Poor adherence to generic recommendations, like PA prescriptions, presents an opportunity for tailoring clinic-based intervention strategies using information about the contexts of patients’ day-to-day routines that are amenable to change. The results from this study have clinical relevance for identifying where, when, and how to support patients with different behavior change goals. Examples of four patient time-use patterns (Figure 4) illustrate how time-use patterns could be applied to enhance intervention strategies within a clinic-based behavior change intervention. We discuss the potential of integrating time-use patterns to strengthen behavior change interventions; however, further research is required to better understand the clinical relevance of time-use patterns for healthcare professionals in different intervention programs and settings. 

The unique contexts of time-use patterns for P01, P06, P18, and P26 show that patients spend significant portions of time sedentary with very little time spent in light PA (Figure 1). The clinical relevance of time-use patterns (Figure 4) is shown using green stars to highlight when and where intervention strategies could be used to disrupt routines and encourage patients with increasing PA, such as breaking up long periods of sedentary activity. For example, time-use patterns for P18 show that after a morning walk in their neighborhood, they are sedentary at home watching television and reading. Knowing that P18 watches the same television show at 10:00 a.m. on weekdays provides context for tailoring specific recommendations, such as encouraging P18 to gradually increase the pace and distance covered without changing the amount of time spent walking. P18 could also be encouraged to adapt sedentary activity between 11:00 a.m. to 12:30 p.m. when they are usually reading. Tailored recommendations could include encouraging P18 to listen to an audiobook while engaging in some form of light to MVPA, like a leisurely walk or housework/labor. Lastly, P18 is largely sedentary at home during the evening after dinner when they are watching television. Integrating some form of light to MVPA, like engaging in stretching and strengthening exercises while watching television, could encourage small incremental changes to established routines. 

Understanding the context surrounding where and when day-to-day routines occur is important for providing tailored recommendations that can be integrated into established routines. For example, patients who spend significant portions of time at home are more likely to adhere to specific recommendations for integrating home-based light PA or MVPA into existing routines versus generic recommendations that require new routines, like going to a recreation facility to engage in PA [21]. The potential opportunities for disrupting routines (Figure 4) emphasize the importance of meeting patients where they are by engaging with patients who are at different stages of readiness to change [57]. 

Visually displaying patients’ time-use sequences provides insight into how data could be organized and stratified so that differences in patient activities can be easily interpreted. Stratifying patients’ time-use patterns by age, sex, employment status, time spent at home or away from home, and level of PA could be powerful for identifying intervention strategies that could be integrated into existing routines for as many patients as possible. For example, understanding how much time patients spend at home or away from home is important for working with heterogeneous patient groups to develop context-specific intervention strategies based on resources of different places and opportune times to disrupt day-to-day routines. For example, information about the way individuals interact with their environment could enhance time-sensitive prompts that encourage switching between tasks, such as breaking up long periods of sedentary activity when patients are watching television in the evening [45].

Time-use patterns characterize where and when day-to-day routines occur; however, contexts influencing who patients are with, such as social influences and social networks, are important for characterizing patterns of physical activity [58] and strengthening interventions to sustain behavior change [59,60]. Research exploring social influences on decision-making processes, such as factors influencing where and with who patients engage in physical activity, could improve knowledge of how to tailor intervention strategies by utilizing supportive social and environmental influences. For example, healthcare professionals can increase social commitments to PA by encouraging patients to schedule PA with family and friends.

In addition to stratifying patient results, different color code schemes could be used to highlight time-use patterns specific to behavior change goals, like medication adherence, diet, or other self-management of chronic illness tasks. For example, identifying where and when patients take medications could inform how interventions could be tailored to improve medication adherence based on knowledge of where and what patients are doing or whether medication times could be adjusted to a more opportune time and place based on patient routines. The color coding schematic chosen for time-use patterns was intended to emphasize levels of PA so that at first glance, a healthcare provider could quickly gather information about the time of day, place, and types of activities that could be adapted to increase PA. 

### 4.2. Strengthening Behavior Change Interventions

Eliciting information on patient spatiotemporal activity is useful for identifying where, when, and how high-risk activities are part of day-to-day routines. Using a customized spatiotemporal data collection application was valuable during interviews to assist patients with recalling details about where, when, and for how long day-to-day activities occur. While this study focused on PA, multiple health-related behaviors are connected in space, place, and time [61]. Most behavior change interventions have focused on single health behaviors [15], and further investigation is required to identify what strategies can improve the tailoring of interventions targeting multiple health behaviors [62]. Geo-ethnographic techniques were useful in this study for identifying the contexts of where, when, and how multiple health activities intersect in space, place, and time. Time-use sequences that were developed in this study could be strengthened by integrating contexts about social relationships and connections to places that influence patients’ day-to-day activities. Expanding on the spatiality of patient activities beyond location and time is important for behavior change interventions to understand why patients choose certain activities, such as who they are with and perceptions of barriers that inhibit PA, like neighborhood safety and walkability. Time-use sequences could also be strengthened by highlighting patients’ social relationships, influencing constraints and opportunities for behavior change. Behavior change interventions commonly focus on educating patients with PA training and nutritional counseling; however, these approaches do not adequately consider the importance of patients’ social relationships, including who prepares meals or who interactions typically occur with. Knowing who patients spend their time with is important for behavior change interventions so that patients’ social networks can be included within intervention strategies and patients can be supported by their social network with maintaining health-improving activities.

The results also contribute to our understanding that overemphasizing one activity, like minimum thresholds for MVPA, may impose unnecessary expectations and barriers for individuals that would benefit from increased activity at lower levels [63,64]. Evidence indicates even small amounts of light PA are associated with a reduced risk of premature mortality and secondary prevention of a repeat event [64,65]. However, messaging around minimum guidelines for daily MVPA could negatively influence patients’ self-awareness about the importance of staying active throughout the day, aside from small portions of MVPA [66]. Visualizing activities on a space-time continuum, we can see it is problematic to overemphasize one activity, like MVPA, without considering the contexts of how patients spend the rest of their day. A visualization of time-use patterns could be useful within clinic-based settings for informing conversations between patient and provider, increasing information available for discussing when and where specific strategies could be adopted to solicit change and improving continuity of care using contexts of day-to-day routines as a guide for monitoring and following up with how patients are adapting their routine activities between appointments. 

Understanding the contexts of opportunities or constraints of patients’ existing routines is important for supporting patients across diverse contexts, especially patients with diverse backgrounds who experience barriers from chronic time deficits. For example, generic intervention strategies that encourage patients to spend more time exercising require existing routines to be disrupted and time to be allocated away from other activities. Developing different routines is challenging and unrealistic for patients constrained by chronic time deficits [16,67]. Behavior change interventions could be strengthened by improving healthcare professionals’ awareness of different time constraints patients face day to day and how constraints of limited time and resources influence automatic versus reflective decisions about day-to-day activities. For example, it is not evident how behavior change interventions are adapted to meet the needs of patients in diverse and marginalized populations who experience unique contextual barriers and time deficits [68]. Geo-ethnography techniques could be a useful tool for exploring when and where patients are challenged with disrupting routines, such as times of the day patients have depleted self-regulating control for switching between activities and commonly rely on automatic decisions about engaging in habitual day-to-day routines. Disrupting routines to develop different habits requires an understanding of time-use patterns to determine when, where, and how different activities could be integrated into a new routine. The use of geo-ethnography contributes knowledge of potential techniques for monitoring changes to patient routines and maintenance of new routines over time [69]. For example, evidence that new habits require a minimum of 6 weeks to form [70,71] suggests a need for behavior change interventions to integrate time-use patterns for monitoring how patients adjust to different habits and maintain new routines beyond 6 weeks. Further research is required to investigate what types of intervention strategies could be implemented to disrupt routines and support patients in developing different health-improving habits and routines that mitigate the risk of chronic disease. 

A strength of this study was using geo-ethnography mixed-method techniques to understand how clinic-based interventions can be modified to gather information about the spatiotemporal contexts that surround patients’ day-to-day routines. The visual reference of a digital map was helpful during interviews for enriching conversations about day-to-day routines and encouraging patients to recall information about the timing and location of their day-to-day activities. An adapted geo-ethnographic approach that integrates participatory techniques could potentially improve the quality and accuracy of spatiotemporal data in comparison to techniques that use survey-based questions. 

Despite the strengths of our approach, there were challenges in customizing the ESRI Field Maps application to capture information about the contexts of every location and activity entered. At the time of the research, it was not feasible to record detailed notes for each location point created during interviews. The primary researcher (BB) reviewed interview transcripts to gather detailed descriptions of each location used to contextualize patients’ day-to-day activities. Furthermore, some participants faced challenges with using the Field Maps application to enter locations and types of activities, requiring the researcher (BB) to assist participants with entering data points (see Appendix A Figure A1). Further research is required to assess the clinical utility of geo-ethnography techniques, such as the feasibility of geo-ethnography surveys prior to patient intake and the clinical relevance of visualization tools in different intervention programs.

The geo-ethnographic approach that was used to develop a visualization tool of patients’ time-use patterns could be automated to facilitate routine clinical use within interventions. For example, information about patients’ time-use patterns could be collected using an online questionnaire guiding patients to fill in details about their typical day-to-day routines, beginning with what time they wake up and activities that occur until they go to bed. Automated outputs of patients’ time-use patterns, like in Figure 1, could be collected at different times throughout an intervention to monitor how patients adapt day-to-day routines and what context-specific behavior change techniques could be used at the most opportune time and place to support patients with individual behavior change goals. 

## 5. Limitations

Although this study contributes knowledge of using spatiotemporal data in the context of a clinic-based secondary behavior change intervention, the patients were from one cardiac program, and their health activities are unlikely to be transferable to larger and more diverse populations at risk of CVD. A limitation of this study was generating static time-use patterns that could quickly be out of date, depending on changes to patients’ day-to-day routines over time. Furthermore, geo-ethnography techniques were time intensive and required lengthy interviews with patients to gather the contexts of spatiotemporal patterns of activity. Automating data entry of patients’ time-use patterns, such as embedding multiple ways patients can interact with a digital application to record their day-to-day routine activities (e.g., voice note recordings, open text notes), could strengthen the integration of geo-ethnography techniques into healthcare settings. Geo-ethnography surveys could be combined with other intake assessments prior to patients being enrolled in clinical interventions, providing additional time for patients to recall details about their day-to-day routines. Gathering this information prior to intake provides an opportunity to optimize the limited time that healthcare professionals have to interact with patients, particularly within clinical settings that have limited human resources. Further research is required to investigate how spatiotemporal-oriented data, like time-use patterns, could be automated within healthcare settings to produce clinically relevant outputs. 

## 6. Conclusions

Improving the effectiveness of clinic-based behavior change interventions requires new approaches to integrate information about spatiotemporal contexts that influence day-to-day routines such as where, when, and how activities occur. This study takes an important first step in developing a tool with the potential to measure and communicate the timing and location of patients’ health activities. Geo-ethnographic techniques are one way of gathering information about spatiotemporal contexts that influence patient day-to-day activities and support the development of stable routines strengthening the maintenance of PA. The results of time-use patterns contribute to understanding potential strategies for modifying secondary interventions to incorporate the contexts of patient activities when recommending interventions. Although this study contributes evidence of techniques for identifying the most opportune time and place to disrupt day-to-day routines, further research is required to explore what strategies would be most effective for encouraging sustained behavior change and modifying activities that reduce CVD risk.

## Figures and Tables

**Table 1:** Data attributes in ESRI Field Maps.

Layer Name	Feature Class	Field ID	Value ID	
Patient ID	Point	ID	P01–P29	
Neighborhood	Polygon	Neighborhood	P01–P29	
Activity	Point	Activity	Walking	Running/Jogging
			Bicycling	Yoga/Stretching
			Strength Training	Playing Sport
			Swimming	Gardening
			Housework	Watching TV
			Cooking/Baking	Shopping
	Point	Food	Grocery Store	Restaurant
			Fast Food	Local Market
			Convenience Store	Specialty Store
			Bar/Brewery	Meal at Home
			Café	
	Point	Social	Family	Friends
	Point	Occupational	Employment	Volunteer
			Caregiver	
	Point	Place of Exercise	Gym Membership	Park
			Community Rec Centre	Home
			Mall/Shopping Centre	Senior Centre
			Outdoor place	Home Condo Facilities
	Point	Healthcare	Healthcare Appointment	Hospital
Transportation	Point	Mode of Transportation	Car	Public Bus
			Walk	Bike
Home	Point	Dwelling Type	House	Condo
			Apartment	

**Table 2:** Description of patient characteristics.

Patient Characteristics	*n*	%
**Age**		
45–54	7	24%
55–64	7	24%
65–74	9	31%
75–84	6	21%
**Sex**		
Male	19	66%
Female	10	34%
**Education Level**		
High school	5	18%
College/trades	12	41%
University/graduate degree	12	41%
**Marital Status**		
Married/common law	22	76%
Single/widow	7	24%
**Household Composition**		
Alone	7	24%
With partner	15	52%
Partner and children	7	24%
**Employment Status**		
Part time	2	7%
Full time	15	52%
Retired	12	41%
**Smoking Status**		
No	14	48.5%
Current smoker	1	3%
Previous smoker	14	48.5%
**Reason for Referral**		
Prevention—no comorbidity	2	7%
Heart attack/stroke—no comorbidity	14	48.5%
Heart attack—comorbidities	13	44.5%

**Table 3:** Levels of activity terms and definitions.

Term	Definition
Sleep (S)	Sleep routine is an important component of health that affects attention, behavior, memory, and overall mental and physical health. Not getting enough quality sleep is linked to a wide variety of health problems, including obesity, type 2 diabetes, cardiovascular disease, and depression [48]. Canadian guidelines recommend getting 7 to 9 h of quality sleep on a regular basis, with consistent bed and wake-up times [48,50].
Sedentary Behavior (SED)	Sedentary behavior is any waking behavior characterized by very low energy expenditure below 1.5 METs (i.e., less than 1.5 times the intensity of rest) while standing, sitting, reclining, or lying down [48,49,50]. Canadian guidelines recommend limiting sedentary time to 8 h or less, including no more than 3 h of recreational screen time and breaking up long periods of sitting as often as possible [48,50].
Light Physical Activity (LT)	Light intensity activities require low levels of energy and effort performed between 1.5 and 3 METs (i.e., greater than 1.5 but less than 3 times the intensity of rest). Light physical activity includes walking at a slower pace, standing work, or light housework [48]. Canadian guidelines recommend getting at least 3 h of light physical activity per day [48].
Moderate-to-Vigorous Physical Activity (MV)	Moderate-to-vigorous physical activity is movement that requires substantial energy expenditure above resting levels performed above 3 METs (i.e., greater than 3 times the intensity of rest) [48]. Moderate-to-vigorous physical activity includes a variety of activities and intensities, like swimming, brisk walking, jogging, rowing, weightlifting, or bicycling. Canadian guidelines recommend participating in at least 30 min per day or 150 min per week of moderate-to-vigorous physical activity [48,50].

**Table 4:** Summary of patients’ typical time-use patterns.

	*n*	Av. Time	
	(m, f)	(Range)	Av.%
Sleep			
≥7 h Minimum Guideline	26	8.25	34%
	(17, 9)	(7–10)	
<7 h Minimum Guideline	3	6.25	26%
	(2, 1)	(6–6.5)	
Total Sleep Activity	29	8	33%
	(19, 10)	(6–10)	
Sedentary Activity			
≥8 h Maximum Guideline	24	12.25	51%
	(17, 7)	(8.5–15.5)	
<8 h Maximum Guideline	5	5.4	22%
	(2, 3)	(1–8)	
Total Sedentary Activity	29	11	46%
	(19, 10)	(1–15.5)	
Employment from Home	6	7	29%
	(3, 3)	(5–9)	
Employment from Worksite	5	8	33%
	(4, 1)	(7–8.5)	
Watching Television	27	3.5	14%
	(18, 9)	(1–8)	
Reading	19	2	8%
	(14, 5)	(0.5–3.5)	
Social Activities	4	1.6	7%
	(3, 1)	(1–2.5)	
Light Physical Activity			
≥3 h Minimum Guideline	14	6.25	26%
	(8, 6)	(4–14)	
<3 h Minimum Guideline	15	1.4	6%
	(11, 4)	(0–2.5)	
Total Light Physical Activity	29	3.7	15%
	(19, 10)	(0–14)	
Employment from Worksite	4	9.75	40%
	(2, 2)	(7–14)	
House Chores/Labor	17	2	8%
	(11, 6)	(1–2.5)	
Moderate-to-Vigorous Physical Activity (MVPA)			
≥30 min Minimum Guideline	24	1.5	6%
	(16, 8)	(0.5–5.5)	
<30 min Minimum Guideline	5	0	0%
	(3, 2)		
Total MVPA	29	1.2	5%
	(19, 10)	(0.5–4.5)	
Walking	22	1.2	5%
	(15, 7)	(0.5–4.5)	
Exercise Class	3	1.5	6%
	(1, 2)	(0.5–2)	
Golf	1	4	16%
	(0, 1)		

## Data Availability

The data that support the findings of this study are not publicly available to ensure the privacy and protection of the participants’ identities. Anonymized and de-identified data may be made available from the corresponding author upon reasonable request.

## Abbreviations

CVD	cardiovascular disease
EMA	ecological momentary assessment
GPS	global positioning system
METs	metabolic equivalents scores
PA	physical activity
MVPA	moderate-to-vigorous physical activity
